# Thermodynamics-Based Model Construction for the Accurate Prediction of Molecular Properties From Partition Coefficients

**DOI:** 10.3389/fchem.2021.737579

**Published:** 2021-09-13

**Authors:** Deliang Chen, Xiaoqing Huang, Yulan Fan

**Affiliations:** Jiangxi Key Laboratory of Organo-Pharmaceutical Chemistry, Chemistry and Chemical Engineering College, Gannan Normal University, Ganzhou, China

**Keywords:** computational chemistry, linear free energy relationships, molecular properties, partition coefficient, noncovalent interactions, quantitative structure-property relationships

## Abstract

Developing models for predicting molecular properties of organic compounds is imperative for drug development and environmental safety; however, development of such models that have high predictive power and are independent of the compounds used is challenging. To overcome the challenges, we used a thermodynamics-based theoretical derivation to construct models for accurately predicting molecular properties. The free energy change that determines a property equals the sum of the free energy changes (ΔG_F_s) caused by the factors affecting the property. By developing or selecting molecular descriptors that are directly proportional to ΔG_F_s, we built a general linear free energy relationship (LFER) for predicting the property with the molecular descriptors as predictive variables. The LFER can be used to construct models for predicting various specific properties from partition coefficients. Validations show that the models constructed according to the LFER have high predictive power and their performance is independent of the compounds used, including the models for the properties having little correlation with partition coefficients. The findings in this study are highly useful for applications in drug development and environmental safety.

## Introduction

The rapid development of new organic compounds in various chemical-related laboratories and industries has increased the difficulty in measuring the physicochemical, and absorption, distribution, metabolism, excretion, and toxicity (ADME/Tox) properties of all possible compounds. Therefore, the development of techniques for predicting these properties via computational tools is imperative (Sarkar et al., 2012; Li et al., 2019; Sun et al., 2019; Suay-Garcia et al., 2020). Quantitative structure–property relationships (QSPRs) with multiple predictive variables are widely used for predicting various properties of organic compounds. QSPR employs regression statistics using algorithms, such as artificial neural networks, (Deeb et al., 2011; Song et al., 2017), machine learning (Bushdid et al., 2018; Cheng and Ng, 2019; Zheng et al., 2019), and partial least square (Deeb et al., 2011; T. Stanton, 2012), with predictive variables usually selected from a few thousand molecular descriptors based on mathematical and statistical tools (Mansouri et al., 2018; Lee et al., 2019; Fioressi et al., 2020). A large number of articles related to QSPR were published per year and QSPR has gained importance in a wide range of fields, such as drug design, pesticide design, and environmental toxicology (Roy et al., 2018; Yang et al., 2018; Zhu et al., 2018; He et al., 2019; Khan et al., 2019; Zhu et al., 2020a). For example, predicting the ADME/Tox of drug candidates before synthesis can significantly reduce the cost and time of drug development and increase the success rate (Cheng et al., 2013; Dickson et al., 2017; Zhu et al., 2018). Predicting soil/water partition coefficients and the toxicities of organic compounds is vital for environmental risk assessments (Freitas et al., 2014; Sabour et al., 2017; Khan et al., 2019). Some properties can be predicted accurately with hydrophobicity (logP_oct_, the logarithm of the partition coefficient between n-octanol and water) and/or other commonly used molecular descriptors, e.g., electrophilicity index (*ω*) (Raevsky, 2004; Pal et al., 2019; Jana et al., 2020). For example, logP_oct_ has been used to predict the water solubility with high accuracy, (Raevsky, 2004), Robust multiple linear regression (MLR) models for toxicity prediction can be constructed by using the combinations of electronic factor (ω, ω^2^, or ω^3^) and hydrophobicity factor [logP_oct_, (logP_oct_)^2^] as predictors (Pal et al., 2019; Jana et al., 2020). The robustness of the models were ascertained by neural networks. However, for many properties, constructing QSPR models with high predictive accuracy and reliability remains a challenge. The performance of QSPR models greatly depends on the compounds used for investigation, quality of the data, and modelling methodology employed (Song et al., 2017; Mansouri et al., 2018; Zhang et al., 2020). For a given property, the predictive variables would be different if the data in the training set are different. In addition, QSAR models usually work well only for the compounds within their applicability domains and do not have good predictive accuracy for other compounds (Kaneko, 2017; Liu and Wallqvist, 2019). However, it is difficult to define the accurate applicability domains for QSPR models because there is no general agreement for quantifying compound similarity (Carrió et al., 2016). It is thus important to develop a new methodology for constructing models that have high predictive power and the performance of the models is independent of the compounds used.

The quantitative formula and quantitative relationships that are developed via theoretical derivation in physical chemistry are absolutely correct and are independent of the compounds used. For example, the partition coefficient between water and an organic solvent (logP_ow_) for a solute is directly proportional to the standard free energy change for transferring the solute from water to the organic solvent (ΔG_tr_). The ΔG_tr_ in turn depends on the standard enthalpy change (ΔH_tr_) and entropy change (ΔS_tr_) of the phase-transferring process. Thus, at a given temperature, the model logP_ow_ = b_1_ΔH_tr_ + b_2_ΔS_tr_ + c (b_1_, b_2_, and c are constants) is absolutely correct and has high predictive power for predicting logP_ow_. This example indicates that the models developed via thermodynamics-based theoretical derivations may overcome the shortages of the models developed by using mathematical and statistical tools. A large number of physicochemical properties, ADME/Tox qualities, and many other properties of organic compounds depend on the changes in free energy caused by the intermolecular noncovalent interactions of the compounds with their environments. The enormous catalytic power of many enzymes depends on the noncovalent interactions between substrates and enzymes (Warshel et al., 2006; Chen et al., 2019). It is thus expected that models with high predictive power for many properties can be developed by considering the free energy changes related to the properties. In this study, we used a thermodynamics-based theoretical derivation to develop a general linear free energy relationship (LFER) for predicting various properties of organic compounds. The LFER can be used to construct models for many specific properties. Validation shows that the models for specific properties have high predictive power and their performance is independent of the compounds used.

## Computational Methods

### Data set selection

In this study, all experimental data of logP_oct_, logP_16_ (the logarithm of the partition coefficient between hexadecane and water), logP_chl_ (the logarithm of the partition coefficient between chloroform and water), logP_aln_ (the logarithm of the partition coefficient between aniline and water), logK_brain_ (the logarithm of the partition coefficient from air to human brain) and logK_p_ **(**logarithm of experimental human skin permeability) are collected from literatures (Abraham et al., 1994; Abraham et al., 1999; Abraham and Martins, 2004; Abraham et al., 2006; Abraham et al., 2015; Zhang et al., 2017). Hydrogen bond acceptors (HBAs) include very weak H-bond acceptors. For example, the sp2 carbon atoms from carbon-carbon double bonds and aromatic rings are weak HBAs. Hydrogen bond donors (HBDs) include very weak H-bond donors. For example, the hydrogen atoms in CHCl_3_ and CH_3_NO_2_ are weak HBDs.

### Calculation of S_m_

S_m_ is a molecular descriptor developed in this study. The S_m_ values of organic compounds were calculated based on the formula of the compounds. Assume the formula of a neutral organic compound is C_c_H_h_O_o_N_n_S_s_F_f_Cl_cl_Br_br_I_i_, the S_m_ of this compound is

$$S_{m}\mathrm{=c+0}\mathrm{.3h+o+n+2s+0}\mathrm{.6*f+1}\mathrm{.8cl+2}\mathrm{.2br+2}\mathrm{.6i-0}\mathrm{.2}N_{\mathrm{c3}}\mathrm{-0}\mathrm{.6}N_{\mathrm{c4}}\text{.}$$ (1)

In Eq. 1, c, h, o, n, s, f, cl, br and i are the numbers of carbon, hydrogen, oxygen, nitrogen, sulfur, fluoride, chloride, bromide and iodide atoms in the solute, N_c3_ is the number of sp3 carbons connecting three heavy atoms (fluoride is not included), N_c4_ is the number of sp3 carbons connecting four heavy atoms (fluoride is not included).

### Calculation of H_M_HBD_

H_M_HBD_ values of solutes were calculated based on the approach reported in a previous study (Chen et al., 2020).

### Calculation of Flexibility

In this study, the flexibility of a solute is calculated by summarizing the flexibilities of the bonds of the solute. If a bond is not rotatable or if the rotation of a bond does not change the conformation of the solute, the flexibility of the bond is set to zero (note: hydrogen atoms are not included for determining conformations). The flexibility of the C—C bond in R^1^CH_2_—CH_2_R^2^ is set to one. If the energy barrier for rotating a bond is obviously higher than that for rotating the R^1^CH2—CH_2_R (Sun et al., 2019) bond, the flex value is set to zero. For example, the C—N bond in RCO—NH and the C—C bond in Ar—CO are set to zero. If the energy barrier for rotating a bond is obviously lower than that for rotating the R^1^CH_2_—CH_2_R^2^ bond, the flex value is set to 1.5. For example, the energy barrier for rotating the R^1^O—CH_2_R^2^ bond is lower than that for R^1^CH_2_—CH_2_R (Sun et al., 2019) and thus the Flex value of the C—O bond is set to 1.5. Also, the flexibility of C—C in R^1^CH_2_—C_6_H_5_ is set to 0.5 because of the symmetry of phenyl ring.

### Calculation of the effects of HBAs on the logP_oct_/logP_chl_

The free energy changes for transferring depolarized solutes from water to hexadecane (ΔG_tr_depol_) were calculated based on the method reported in previous study (Chen et al., 2020). Based on the logP_oct_ (or logP_chl_) and ΔG_tr_depol_ values of nonpolar compounds, the model for the regression of logP_oct_ (or logP_chl_) against ΔG_tr_depol_ was developed. This model was then used to calculate the logP_oct_ (or logP_chl_) values for depolarized solutes*.* For a solute containing HBAs but no HBDs, the difference between the calculated logP_oct_ (or logP_chl_) for the depolarized solute and the experimental logP_oct_ (or logP_chl_) of the solute is the effect of HBAs on the logP_oct_ (or logP_chl_) of the solute.

### Model development

All the models and the statistical reliabilities of the models were obtained by performing the multiple linear regressions implemented in Excel.

## Results and Discussion

### Thermodynamics-Based Theoretical Derivation for Generating a Linear Free Energy Relationship

In the theoretical derivation, we used “Y” to represent a property and the symbol “ΔG_Y_” to represent the free energy change that determines Y. The ΔG_Y_ values for many properties are not easy to be calculated directly. Thus, we decomposed ΔG_Y_ into the free energy changes that are caused by the factors affecting Y. The free energy change caused by a factor is denoted by “ΔG_F_”. Thus, ΔG_Y_ equals the summarization of the ΔG_F_s for all the factors affecting Y.

$$\Delta G_{Y}=\sum \Delta G_{F}$$ (2)

For the properties depending on the noncovalent interactions of solutes, they are affected by the molecular sizes, hydrogen-bond acceptors (HBAs), and hydrogen-bond donors (HBDs) of the solutes, which was demonstrated in a previous study (Chen et al., 2020). Many properties are also affected by the flexibilities of solutes. For example, the partition coefficients of organic compounds between a flexible environment (e.g., blood) and a much less flexible environment (e.g., muscle) are obviously affected by the flexibilities of the compounds. It is challenging to accurately quantify the ΔG_F_s for various properties. However, it is possible to develop molecular descriptors that are directly proportional to ΔG_F_s. We used D_F_ to represent the molecular descriptor that is directly proportional to ΔG_F_. Then, ΔG_Y_ can be expressed as:

$$\Delta G_{Y}=\sum k_{F}D_{F}$$ (3)

The k_F_ values are constant for a given property. Theoretically, if the molecular descriptors apply to various properties, Eq. 3 can be used to construct models with high predictive power for the properties. Many properties are mainly affected by the molecular sizes, HBAs, HBDs and flexibilities of solutes. Thus, in this study, we developed or selected molecular descriptors for quantifying the effects of molecular size, HBAs, HBDs and flexibility on the properties.

**FIGURE 1:**
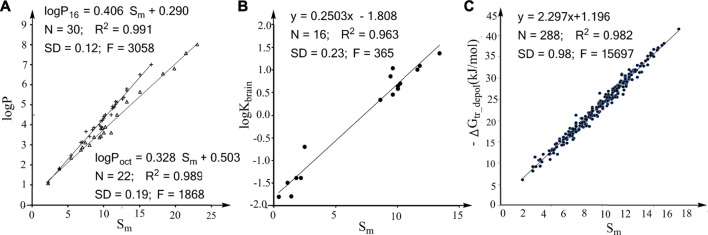
Correlations between the molecular descriptor S_m_ and the effects of molecular size on various properties. **(A)** Linear associations between logP_16_/logP_oct_ and S_m_ for alkanes. **(B)** Linear association between logK_brain_ (log of the partition coefficient from air to human brain) and S_m_ for nonpolar compounds. **(C)** Plot of the water to hexadecane phase-transferring free energy for depolarized solutes (ΔG_tr_depol_) against the S_m_ values of the solutes.

The molecular descriptor we developed for quantifying the effects of molecular size on properties is denoted by “S_m_”. The S_m_ values of organic compounds represent the relative molecular sizes of the compounds and can be easily calculated from their molecular formulas (see Computational Methods). For example, the S_m_ for catechol (formula: C_6_H_6_O_2_) is 9.8 (num. for C + 0.3× num. for H + num. for O). To illustrate whether S_m_ is an ideal molecular descriptor for molecular size, we first explored the linear associations between logP_16_ and S_m_ and between logP_oct_ and S_m_ for a series of alkane compounds (Figure 1A). The logP_16_ and logP_oct_ values for alkane compounds are affected merely by the sizes of the compounds. The robust linear associations in Figure 1A support that S_m_ is directly proportional to the effects of molecular size on logP_16_ and logP_oct_. We next explore whether S_m_ is also an ideal molecular descriptor of molecular size for the properties that have little correlation with logP_16_ or logP_oct_. As reported in a previous study, logK_brain_ has little correlation with logP_16_ and logP_oct_ (Chen et al., 2020). We thus explored the linear association between logK_brain_ and S_m_ for nonpolar solutes (Figure 1B). The R^2^ and SD values indicate that there is a strong linear association between logK_brain_ and S_m_. In Figure 1C, we plotted the free energy changes for transferring the depolarized compounds from water to hexadecane (ΔG_tr_depol_) against the S_m_ values for the compounds from Supplementary Table S1 of a previous study (Chen et al., 2020). The high statistical reliability for the regression of ΔG_tr_depol_ against S_m_ further supports that S_m_ is an ideal molecular descriptor for quantifying the effect of molecular size on the properties depending on noncovalent interactions. Thus, S_m_ is an ideal molecular descriptor for molecular size and applies to various properties.

**FIGURE 2:**
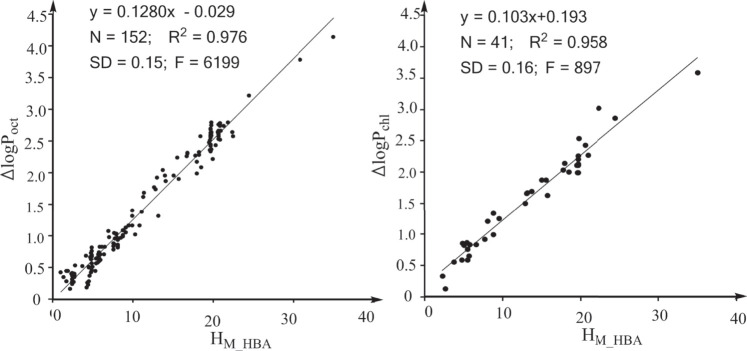
Strong linear associations between the effects of HBAs on properties and HM_HBA. H_M_HBA_: overall H-bonding capabilities of the HBAs of a solute. **(A)** For the property logP_oct_ (log of the partition coefficient between n-octanol and water). **(B)** For the property logP_chl_ (log of the partition coefficient between chloroform and water).

In previous studies, we defined the water to hexadecane phase transferring free energy contributed by the electrostatic interactions of the HBAs of a solute as the overall H-bonding capability of the HBAs of the solute (Chen et al., 2019; Chen et al., 2020; Chen et al., 2016) and this overall H-bonding capability is donated by “H_M_HBA_.” The definition indicates that H_M_HBA_ is an ideal molecular descriptor for quantifying the effects of HBAs on logP_16_. We next explored whether H_M_HBA_ is an ideal molecular descriptor for logP_oct_ and logP_chl_. The strong linear associations between the effect of HBAs on logP_oct_ and H_M_HBA_ (Figure 2A) and between the effect of HBAs on logP_chl_ and H_M_HBA_ (Figure 2B) suggest that H_M_HBA_ is an ideal molecular descriptor for quantifying the effects of HBAs on various properties. Similarly, we defined the water to hexadecane phase transferring free energy contributed by the electrostatic interactions of the HBDs of a solute as the overall H-bonding capability of the HBDs of the solute (H_M_HBD_) (Chen et al., 2016; Chen et al., 2019; Chen et al., 2020). In a previous study, we revealed that the contribution of a protein-ligand H-bond to the protein-ligand binding free energy is directly proportional to the H-bonding capability of the HBA and the H-bonding capability of the HBD (Chen et al., 2016). We also found that the effect of an enzyme-substrate H-bond interaction on the free energy barrier of the enzymatic reaction is directly proportional to the H-bonding capability of the atom from the enzyme (Chen et al., 2019). Thus, we believe that the effects of HBAs and HBDs of solutes on the properties related to noncovalent interactions are directly proportional to the H_M_HBA_ and H_M_HBD_ values of the solutes. H_M_HBA_ and H_M_HBD_ are ideal molecular descriptors for quantifying the effect of HBAs and HBDs on the properties related to noncovalent interactions.

The molecular descriptor for quantifying the effect of molecular flexibility on properties is denoted by “Flex.” The effects of molecular flexibility on properties mainly result from rotatable bonds of the solutes because the rotatable bonds of the solutes can rotate more freely in some environments than in other environments. The flexibilities of solutes are calculated from the rotatable bonds of the solutes, especially the rotatable bonds that change the conformations of solutes (see Computational Methods). Thus, for many properties that are affected by molecular size, HBAs, HBDs and flexibility, they can be quantified with the following equation

$$\mathrm{Y=}k_{1}S_{m}+k_{2}\;H_{\mathrm{M\_HBA}}+k_{3}H_{\mathrm{M\_HBD}}+k_{4}\mathrm{Flex+c}$$ (4)

where k_1_, k_2_, k_3_, k_4_ and c are constants for a give property. Organic compounds usually contain multiple HBAs and the HBAs affect each other. The accurate calculation of H_M_HBA_ for many organic compounds is not easy. Eq. 4 would become simpler and easier to use if H_M_HBA_ is replaced by logP_ow_ because logP_ow_ is a well-known molecular descriptor for predicting properties (Liu et al., 2019; Zhu et al., 2020b) and can be obtained accurately via experimental and/or computational approaches. Based on the fact that logP_ow_ is a property and Eq. 4 also applies to logP_ow_, we can convert Eqs. 4 to 5 (see Supplementary Text S1 for the detail of the process of the conversion).

$$\mathrm{Y=}b_{1}\log P_{\mathrm{ow}}+b_{2}S_{m}+b_{3}H_{\mathrm{M\_HBD}}+b_{4}\mathrm{Flex+c}$$ (5)

where b_1_, b_2_, b_3_, and b_4_ are constants, logP_ow_ is logP_16_ or logP_oct_. Eq. 5 is identical to Eq. 4. Both equations are correct for the properties that are determined by the noncovalent interactions of solutes with flexible environments. All the factors related to effects of noncovalent interactions on phase-transferring free energies, including electrostatic interaction, desovation, van der Waals interactions, entropy change, etc. are considered in Eq. 5. Eq. 5 is the general LFER we developed for predicting the properties that depends on the noncovalent interactions of solutes with flexible environments. Although S_m_ and H_M_HBD_ may be strongly correlated with logP_ow_ for some properties, none of the molecular descriptors can be omitted because Eq. 4 is a general LFER for various different properties.

### Validation of the General LFER: Model Construction for Specific Properties

#### Prediction of Various Organic Solvent/Water Partition Coefficients

**FIGURE 3:**
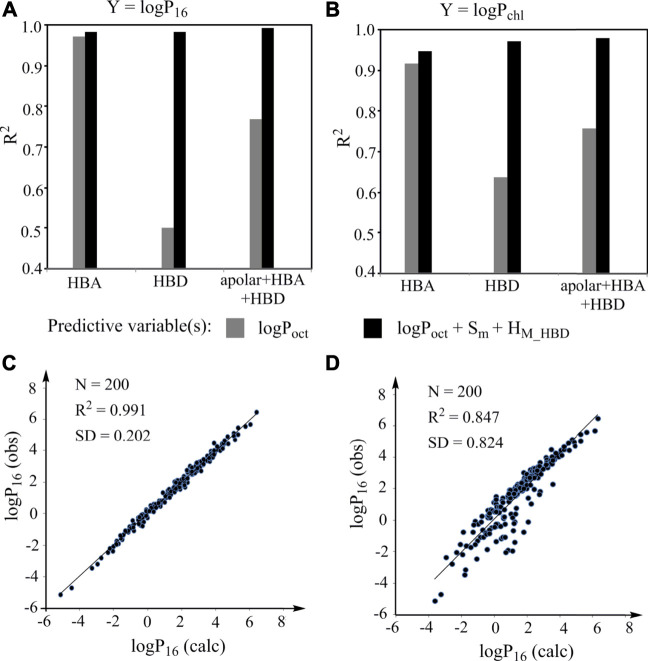
Prediction of organic solvent/water partition coefficients for validating the general LFER. **(A, B)** R^2^ values of the simple regressions (gray columns) of logP_16_(A)/logP_chl_(B) against logP_oct_ and of the corresponding models constructed according to the LFER (black columns). HBA: compounds containing HBAs but no HBDs; HBD: compounds containing HBDs; apolar: nonpolar compounds; **(C)** Plot of observed logP_16_ against the logP_16_ calculated from the model constructed according to the LFER; **(D)** Plot of observed logP_16_ against the logP_16_ calculated from the model with logP_oct_ as predictive valuable.

To prove that this general LFER can be used to construct models with high predictive power for various specific properties, we first demonstrated that it can be used to predict an organic solvent/water partition coefficient from another organic solvent/water partition coefficient with high accuracy. Eighty-nine compounds with experimental logP_oct_, logP_16_, and logP_chl_ values (Abraham et al., 1994; Abraham et al., 1999) (Supplementary Table S1) were used for this investigation. Among the compounds, 45 compounds contain HBAs but no HBDs and 41 compounds containing HBDs. The equations and statistical results of the simple regressions of logP_16_ against logP_oct_ and logP_chl_ against logP_oct_ for various types of compounds are shown in Supplementary Text S2. The R^2^ (squared correlation coefficient) values of the regressions range from 0.501 to 0.972 (gray columns, Figures 3A,B) and the SD (standard deviation) values of the regressions range from 0.241 to 0.965, indicating that the strength of the linear associations between two partition coefficients largely depends on compounds used for investigation. Then the same data for constructing the simple regressions were used to construct models according to the general LFER and the results are also shown in Supplementary Text S2 (note: the model descriptor Flex is not used because Flex has little effect on logP_ow_). The R^2^ values of the models range from 0.947 to 0.992 (black columns, Figures 3A,B) and the SD values range from 0.183 to 0.248. The results indicate that the models constructed according to the LFER have a high statistical reliability and the performance of the models is independent of the compounds for investigation.

To demonstrate whether the models have high predictive power, we compared the experimental logP_16_ values of 200 organic compounds [from Supplementary Table S1 of a previous study (Chen et al., 2020)] and the logP_16_ values calculated from the model constructed according to the LFER by using an external validation approach (Figure 3C and Supplementary Text S3). The result shows that the model has high predictive power. For comparison, the predictive power of the corresponding simple regression was also investigated (Figure 3D), which is much worse than that for the model constructed according to the LFER. Thus, the LFER is powerful for constructing models with high predictive power.

#### Prediction of the Human Skin Permeability

We next used the LFER to construct a model for predicting the human skin permeability of neutral organic molecules. Supplementary Table S2 shows the logK_p_ (Abraham and Martins, 2004; Zhang et al., 2017) values of 51 organic compounds. Thirty-two of the compounds were used as training set to develop the model with logP_oct_ as a predictive valuable and the other 19 compounds as a test set to validate the model. The model constructed according to the LFER is shown below.

$$\begin{array}{l} \log K_{p}\mathrm{=0}\mathrm{.6157log}P_{\mathrm{oct}}\mathrm{+0}\mathrm{.0156}S_{m}\mathrm{-0}\mathrm{.0626}H_{\mathrm{M\_HBD}}\mathrm{-0}\mathrm{.0988Flex-5}\mathrm{.646;}\\ \mathrm{N=32,}R^{2}\mathrm{=0}\mathrm{.953,}Q^{2}\left(\mathrm{ext}\right)\mathrm{=0}\mathrm{.966,SD=0}\mathrm{.178;F=136}\mathrm{.7} \end{array}$$ (6)

**FIGURE 4:**
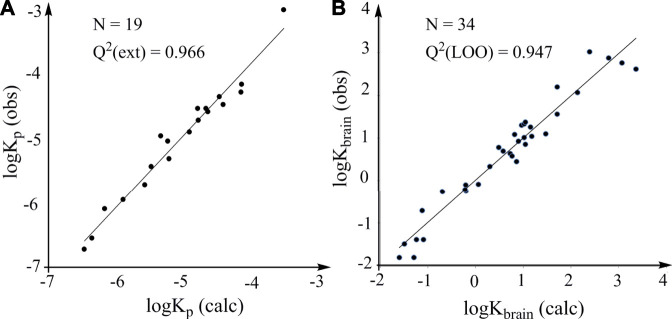
Predictive power of models constructed according to the LFER. **(A)** External validation. Plots of observed logK_p_ (log of human skin permeability) against the logK_p_ calculated from the model constructed according to the LFER (logP_oct_ is used). **(B)** LOO cross-validation. Plots of observed logK_brain_ against the logK_brain_ calculated from the model constructed according to the LFER.

This model is characterized by high statistical reliability according to the R^2^ and SD values. It is used to calculate the logK_p_ values of the 19 solutes in the test set. The plot of the experimental logK_p_ values versus the calculated logK_p_ values is characterized by statistically robust linearity (Figure 4A). The accurate prediction of logK_p_ can provide a rapid and accurate prediction of human skin permeability of organic compounds, which is very useful for evaluating environmental risks due to contact with skin.

#### Prediction of Air to Human Brain Partition Coefficient

To further illustrate the reliability and accuracy of the LFER, we used the LFER to construct models for the properties that have little correlation with logP_ow_. The strength of the linear association between logK_brain_ and logP_oct_ (or logP_16_) is weak (R^2^ < 0.1) (Chen et al., 2020). Supplementary Table S3 lists the compounds that were used to demonstrate the weak linear association between logK_brain_ and logP_16_ (or logP_oct_) in a previous study (Chen et al., 2020). Based on the experimental logK_brain_, logP_16_ and logP_oct_ data of the compounds, we constructed two models according to the LFER.

$$\begin{array}{l} \log K_{\mathrm{brain}}\mathrm{= -0}\mathrm{.5129log}P_{\mathrm{16}}\mathrm{+0}\mathrm{.5006}S_{m}\mathrm{+0}\mathrm{.1009}H_{\mathrm{M\_HBD}}\mathrm{-0}\mathrm{.1893Flex-1}\mathrm{.64;}\\ \mathrm{N=34,}R^{2}\mathrm{=0}\mathrm{.964,}Q_{\mathrm{LOO}}^{2}\mathrm{=0}\mathrm{.947;SD=0}\mathrm{.265;F=195}\mathrm{.7} \end{array}$$ (7)

$$\begin{array}{l} \log K_{\mathrm{brain}}\mathrm{=- 0}\mathrm{.7755log}P_{\mathrm{oct}}\mathrm{+0}\mathrm{.5473}S_{m}\mathrm{+0}\mathrm{.1790}H_{\mathrm{M\_HBD}}\mathrm{-0}\mathrm{.0986Flex-1}\mathrm{.386}\\ \mathrm{N=31,}R^{2}\mathrm{=0}\mathrm{.931,}Q_{\mathrm{LOO}}^{2}\mathrm{=0}\mathrm{.914;SD=0}\mathrm{.368;F=87}\mathrm{.4} \end{array}$$ (8)

Results indicate that both models have high predictive power and the model with logP_16_ as predictive variable is better than the model with logP_oct_ as predictive variable. The predicted logK_brain_ obtained from the LOO cross-validation (logP_16_ is used) and the observed logK_brain_ show a robust linear association (Figure 4B). Thus, the general LFER works well for the properties that have little correlation with partition coefficients, supporting the reliability and efficacy of the general LFER in the accurate prediction of the properties related to noncovalent interactions. We believe that the thermodynamics-based theoretical derivation is a powerful methodology for developing robust models and will be useful in many fields, including drug design, environmental safety and human health.

#### Model Simplification

In some cases, not all the molecular descriptors in the LFER are required for specific models with high predictive power. Some models still have high predictive power without using the molecular descriptor H_M_HBD_. For example, if the HBAs and HBDs of an organic solvent are obviously weaker than the HBAs and HBDs of water, the partition coefficient between water and the organic solvent can be predicted accurately from the model with logP_16_ and S_m_ as predictive variables. Eq. 9 is the model for predicting the aniline/water partition coefficient (logP_aln_) with logP_16_ and S_m_ as predictive variables (see Supplementary Table S4 for the data). Its statistical reliability is high and is obviously better than that for the simple regression (Eq. 10).

$$\log P_{\mathrm{aln}}\mathrm{=0}\mathrm{.4695log}P_{\mathrm{16}}\mathrm{+0}\mathrm{.1506}S_{m}\mathrm{+0}\mathrm{.010;N=54,}R^{2}\mathrm{=0}\mathrm{.975,SD=0}\mathrm{.208,F=1008}.$$ (9)

$$\log P_{\mathrm{aln}}\mathrm{=0}\mathrm{.6416log}P_{\mathrm{16}}\mathrm{+0}\mathrm{.726;N=54;}R^{2}\mathrm{=0}\mathrm{.910;SD=0}\mathrm{.394,F=524}\mathrm{.9}.$$ (10)

Without using H_M_HBD_, the model for predicting logK_brain_ from logP_16_, S_m_, and Flex still has high predictive power.

$$\begin{array}{l} \log K_{\mathrm{brain}}\mathrm{=-0}\mathrm{.6194log}P_{\mathrm{16}}\mathrm{+0}\mathrm{.5446}S_{m}\mathrm{-0}\mathrm{.1928Flex-1}\mathrm{.637;}\\ \mathrm{N = 34,}R^{2}\mathrm{=0}\mathrm{.954,}Q_{\mathrm{LOO}}^{2}\mathrm{=0}\mathrm{.938;SD=0}\mathrm{.295;F=207}\mathrm{.7} \end{array}$$ (11)

Because the calculation of S_m_ and Flex is easy, the accurate prediction of some properties from logP_16_ or logP_oct_ is easy for the researchers across various fields. For example, logK_brain_ can be accurately predicted from logP_16_, without the need for additional experimental data or complicated calculations. Without using the LFER, the accurate prediction of logK_brain_ from logP_16_ or another organic solvent/water partition coefficient is difficult because there is little correlation between logK_brain_ and organic solvent/water partition coefficients. For the models containing H_M_HBD_, the H_M_HBD_ values of solutes are calculated with computer software. All the molecular descriptors in the LFER are easy to be understood and used by the researchers in various research fields. However, when constructing QSPR models by using mathematical and statistical tools, the predictive variables are usually selected from a few thousand molecular descriptors. The meanings of many predictive variables, e.g., the 3D-MoRSE descriptors, (Zapadka et al., 2019), are not easy to be understood or used by many researchers in various research fields.

### Performance of Models With all Molecular Descriptors Calculated From Solute Structures

Because logP_oct_ and logP_16_ can be calculated accurately from the structures of solutes (Chen et al., 2020), it is expected that this method still performs well when all of the molecular descriptors in the LFER are calculated from solute structures. For example, the R^2^, Q^2^_ext_ and SD of the model for predicting human skin permeability, in which all predictive variables are calculated from solute structures, are 0.940, 0.957, and 0.202 (see Supplementary Text S4). Thus, the general LFER developed in this study has obvious advantages in predicting many properties related to noncovalent interactions.

### Importance of Thermodynamics-Based Theoretical Derivation

Above examples indicate that the models constructed according to the LFER for many specific properties have high predictive power. Moreover, the performance of the models is independent of the compounds for investigation, suggesting that the models can provide guidance for improving properties of organic compounds and designing compounds with optimal properties. The merits of the LFER result from the theoretical derivation, which ensures that the quantitative relationships in the models constructed according to the LFER are correct in the aspect of thermodynamics. For the QSPR models developed using mathematical and statistical tools, the predictive variables are selected from a few thousand molecular descriptors based on the data of the compounds in training sets. The relationships between the properties and molecular descriptors in the QSPR models are statistical relationships for the compounds in training sets. The QSPR models usually work well only for the compounds in the training set and similar compounds, but may do not work well for other compounds. Thus, for the properties determined by the noncovalent interactions of solutes with flexible environments, the models developed according to the proposed LFER performs better than the QSPR models developed by using mathematical and statistical tools, including robust artificial neural networks. Developing models according to the proposed LFER is faster and computationally cheap than developing traditional QSPR models because the process of the variable selection is not required. Moreover, the proposed LFER is quite simple and can be easily used by the researchers across various fields, while expert knowledge is required for developing robust artificial neural networks, such as the knowledge in choosing the most appropriate approach. Thus, the method developed in study has obvious advantages over the traditional QSPR construction method. Thermodynamics-based theoretical derivation can be used to solve many problems that are hard to be solved by using mathematical and statistical tools. In addition, results in this study demonstrate that there are quantitative relationships between the properties related to thermodynamics, suggesting that many properties can be accurately predicted from other properties.

### Future Works

The theoretical derivation in this study is based on the assumption that solutes have similar interactions with their environments, which requires that the environments for the properties are flexible or the properties have little relationship with the conformation or orientations of solutes. Thus, the present LFER may not work well in predicting the binding affinities of ligands because the binding sites of proteins are not flexible. If the environment for a property is rigid (e.g., the binding sites of proteins), the model for predicting the property should consider H-bond interactions individually, rather than the overall H-bond interactions. In our further study, we will explore how to develop models for the properties related to rigid environments, which can be used to develop scoring functions for predicting protein-ligand binding affinities and develop QSAR models for screening databases of ligands. Furthermore, in this study, we demonstrated to effectiveness of the LFER for predicting the properties of neutral organic compounds. If a dataset contains ionizable compounds, it will be necessary to include molecular descriptors for the ionized forms. Although several approaches currently exist for considering the effects of ionization on various molecular properties (Li et al., 2006; Zhang et al., 2017), our future work with involve attempts to adapt the proposed LFER for use in these situations.

## Conclusion

In this study, we used a thermodynamics-based theoretical derivation to develop a general LFER for accurately predicting various properties from partition coefficients. The theoretical derivation ensures that many specific properties can be accurately quantified with the molecular descriptors in the LFER. It overcomes the shortages of constructing QSPR models by using mathematical and statistical tools. It is expected that the thermodynamics-based theoretical derivation can be used to solve many difficult problems, including the accurate prediction of protein-ligand binding affinities.

## Data Availability

The original contributions presented in the study are included in the article/Supplementary Material, further inquiries can be directed to the corresponding author.
