# Supplementary material for: Thermodynamics-Based Model Construction for the Accurate Prediction of Molecular Properties From Partition Coefficients
Source: Front Chem. 2021 Sep 13;9:737579. doi: 10.3389/fchem.2021.737579 (PMC8473701; doi:10.3389/fchem.2021.737579)
Supplement: Supplementary file 1 [file DataSheet1.docx]

Supplementary Material

Thermodynamics-based model construction for the accurate prediction of molecular properties from partition coefficients

**Supplementary Text 1: Development of the linear free energy relationship for predicting properties from organic solvent/water partition coefficients.** The following equation is Equation (3) of the main manuscript, which is the model for predicting a property (Y) that is affected by molecular size, HBAs, HBDs and flexibility.

Y = k_1_ S_m_ + k_2_ H_M_HBA_ + k_3_ H_M_HBD_ + k_4_ Flex + c_1_ (S1).

where S_m_ is a molecular descriptor for molecular size, H_M_HBA_ and H_M_HBD_ are the overall H-bonding capabilities of the H-bond acceptors and the H-bond donors of a solute, Flex is solute flexibility, k_1_, k_2_, k_3_, k_4_, c_1_ are constants. For an organic solvent/water partition coefficient (logP_ow_), it is slightly affected by Flex and can be expressed with the following equation.

logP_ow_ = k_5_ S_m_ + k_6_ H_M_HBA_ + k_7_ H_M_HBD_ + c_2_; (S2)

where k_5_, k_6_, k_7_ and c_2_ are constants. Equation (S3) can be derived from Equation (S2).

H_M_HBA_ = (logP_ow_ − k_5_ S_m_ − k_7_ H_M_HBD_ − c_2_)/k_6_  (S3)

On the basis of Equations (S1&S3), the relationship between Y and logP_ow_ can be expressed with Equation (S4)

Y = (k_1_ – k_2_k_5_/ k_6_) S_m_ + (k_2_/ k_6_) logP_ow_ + (k_3_ – k_2_ k_7_/ k_6_) H_M_HBA_ + k_4_ Flex + c_1_ − c_2_ k_3_/k_6_

= b_1_ logP_ow_ + b_2_ S_m_ + b_3_ H_M_HBA_ + k_4_ Flex + c (S4)

where b_1_, b_2_, b_3_ and c are constants. Equation (S4) is the linear free energy relationship for predicting properties from organic solvent/water partition coefficients.

**Supplementary Text 2: Models for predicting an organic solvent/water partition coefficient from *n*-octanol/water partition coefficient.**

A: Simple regression models derived from the data of the 45 HBA compounds in Supplementary Table 1:

logP_16_ = 1.2519(±0.0325)logP_oct_ − 0.731(±0.069);

N = 45, R^2^ = 0.972, SD = 0.241, F = 1482 (S5).

logP_chl_ = 0.9045(±0.0414) logP_oct_ + 0.881(±0.088);

N = 45, R^2^ = 0.917; SD = 0.307, F = 476 (S6).

B. Models derived from the data of the 45 HBA compounds in Supplementary Table 1 with a partition coefficient and S_m_ as predictive variables:

logP_16_ = 1.3365(±0.0302)logP_oct_ − 0.0726(±0.0138)S_m_ − 0.193(±0.116);

N = 45, R^2^ = 0.983, SD = 0.189, F = 1215 (S7).

logP_chl_ = 0.8016(±0.0396) logP_oct_ + 0.0883(±0.0181) S_m_ + 0.338(±0.152);

N = 45, R^2^ = 0.947; SD = 0.248, F = 376 (S8).

C: Simple regression models derived from the data of the 41 HBD compounds in Supplementary Table 1:

logP_16_ = 0.9514(±0.1520)logP_oct_ − 1.757(±0.209);

N = 41, R^2^ = 0.501, SD = 0.965, F = 39.2 (S9).

logP_chl_= 0.8692(±0.1052)logP_oct_ − 0.313(±0.145);

N = 41, R^2^ = 0.637, SD = 0.667, F = 68.3 (S10).

D: Models derived from the data of the 41 HBD compounds in Supplementary Table 1 with a partition coefficient, S_m_ and H_M_HBD_ as predictive variables

logP_16_ = 1.3651(±0.0535)logP_oct_ − 0.0591(±0.0235)S_m_ − 0.2041(±0.0079)H_M_HBD_

− 0.2355(0.129); N = 41, R^2^ = 0.983, SD = 0.184, F = 705 (S11).

logP_chl_=1.0206(±0.0566)logP_oct_+ 0.0287(±0.0249)S_m_ −0.1502(±0.0083)H_M_HBD_

+ 0.377(0.136); N = 41, R^2^ = 0.971, SD = 0.194, F = 410 (S12).

E: Simple regression models derived from the data of the 89 compounds (containing nonpolar, HBA and HBD compounds) in Supplementary Table 1:

logP_16_ =1.3939(±0.0821)logP_oct_ − 1.542(±0.160);

N = 89, R^2^ = 0.768, SD = 0.953,F = 288 (S13).

logP_chl_ =1.1305(±0.0685)logP_16_ + 0.041(±0.133);

N = 89, R^2^ = 0.758, SD = 0.795,F = 272 (S14).

F: Models derived from the data of the 89 compounds (containing nonpolar, HBA and HBD compounds) in Supplementary Table 1 with a partition coefficient, S_m_ and H_M_HBD_ as predictive variables

logP_16_= 1.346(±0.0231)logP_oct_ − 0.1958(±0.0044)H_M_HBD_ – 0.0700(±0.0107) S_m_ – 0.200(±0.076);

N = 89, R^2^ = 0.992, SD = 0.183, F =3365. (S15)

logP_chl_=0.8698(±0.0302)logP_oct_+0.0878(±0.0140)S_m_−0.1688(±0.0057)H_M_HBD_ +0.214(±0.100);

N = 89, R^2^ = 0.979, SD = 0.240, F = 1291 (S16)

**Supplementary Text 3: Predictive power of the model for logP_16_ constructed according to the LFER.** The 89 compounds shown in Supplementary table 1 are used as training set for generating models. The compounds that have both experimental logP_16_ and logP_oct_ values in Supplementary Table 1 of a previous study([Chen et al., 2020](#_ENREF_6)) and are not in the training set and do not have intramolecular H-bonds are used as the test set. There are 200 compounds in the test set.

*Model generation:* A three-variable model with logP_oct_, H_M_HBD_ and S_m_ as predictive valuables was generated from the data of the compounds in the training set. This model is Equation (S15). For comparison, the simple regression of logP_16_ against logP_oct_ is also generated from the same data, which is Equation (S13).

*Model validation:* Equations (S13) and (S15) were used to calculate the logP_16_ values of the 200 compounds in the test set. The relationships between the experimental and calculated logP_16_ values are shown below:

logP_16_ calculated from Equation (S13):

logP_16_ (obs) = 1.0471(±0.0316) logP_16_ (calc) + 0.060(±0.074);

N = 200, R^2^ = 0.848, SD = 0.824, F =1101 (S17).

logP_16_ calculated from Equation (S15):

logP_16_ (obs) = 0.9950(±0.0068)logP_16_(calc) + 0.043(±0.018);

N = 200, Q^2^_ext_ = 0.991, SD= 0.202， F= 21350. (S18)

**Supplementary Text 4: Models for predicting the human skin permeability with the predictive variables calculated from solute structures.** The logK_p_ values of 51 organic compounds listed in Supplementary Table 2 are used to investigate the performance of the LFER model when all predictive variables are calculated from the structures of solutes. The logP_oct_ values are calculated from the structures of the solutes based on the model developed in a previous study([Chen et al., 2020](#_ENREF_6)) and are shown in Supplementary Tables 2. By using the same training and test sets for Equation (5) in the manuscript, we obtain a model for predicting logK_p_, which is shown in Equation (S19). The result of external validation is shown in Equation (S20). It indicates that the LFER model developed in this study still performs well when all predictive variables are calculated from the structures of solutes.

logK_p_ = 0.6117 (±0.0530) logP_oct_ + 0.0131(±0.0177)S_m_ – 0.0561 (±0.0079)H_M_HBD_

– 0.1028(±0.0199)*Flex – 5.645(±0.136);

N = 32, R^2^ = 0.940, SD = 0.202; F = 105 (S19).

logK_p_ (obs) = 1.2052(±0.0620)logK_p_ (clac) + 1.125(±0.318);

N = 19; R^2^ = 0.957, SD = 0.193, F = 378 (S20).

**Supplementary Table 1:** Experimental logP_16_, logP_chl_ and logP_oct_ data of the neutral organic compounds^*^ and the calculated S_m_ and H_M_HBD_ values of the compounds

| Solute | Type* | logP_16_ | logP_chl_ | logP_oct_ | | S_m_ | | H_M_HBD_ |
| --- | --- | --- | --- | --- | --- | --- | --- | --- |
| cyclohexane | apolar | 3.91 | 4.16 | | 3.44 | | 9.6 | 0.00 |
| n-hexane | apolar | 4.49 | 4.69 | | 3.90 | | 10.2 | 0.00 |
| n-octane | apolar | 5.79 | 6.01 | | 5.15 | | 13.4 | 0.00 |
| tetrachloromethane | HBA | 2.88 | 3.31 | | 2.83 | | 7.6 | 0.00 |
| 1,4-dichlorobenzene | HBA | 3.70 | 3.89 | | 3.44 | | 10.8 | 0.00 |
| 1,3-dichlorobenzene | HBA | 3.69 | 3.87 | | 3.53 | | 10.8 | 0.00 |
| iodobenzene | HBA | 3.22 | 3.57 | | 3.25 | | 10.1 | 0.00 |
| chlorobenzene | HBA | 2.84 | 3.40 | | 2.89 | | 9.3 | 0.00 |
| bromobenzene | HBA | 2.97 | 3.63 | | 2.99 | | 9.7 | 0.00 |
| benzene | HBA | 2.15 | 2.76 | | 2.13 | | 7.8 | 0.00 |
| methylbenzene | HBA | 2.68 | 3.41 | | 2.73 | | 9.4 | 0.00 |
| 1,2-dimethylbenzene | HBA | 3.28 | 3.91 | | 3.12 | | 11.0 | 0.00 |
| ethylbenzene | HBA | 3.20 | 3.70 | | 3.15 | | 11.0 | 0.00 |
| 1,3-dimethylbenzene | HBA | 3.23 | 3.68 | | 3.20 | | 11.0 | 0.00 |
| naphthalene | HBA | 3.41 | 4.05 | | 3.30 | | 12.4 | 0.00 |
| diPhenyl | HBA | 4.08 | 4.67 | | 4.06 | | 15.0 | 0.00 |
| diethyl ether | HBA | 0.85 | 1.88 | | 0.89 | | 8.0 | 0.00 |
| 2-nitrotoluene | HBA | 2.25 | 3.39 | | 2.30 | | 12.1 | 0.00 |
| nitrobenzene | HBA | 1.54 | 2.69 | | 1.85 | | 10.5 | 0.00 |
| tetrahydropyran | HBA | 0.77 | 1.99 | | 0.95 | | 9.0 | 0.00 |
| tetrahydrofuran | HBA | 0.09 | 1.31 | | 0.46 | | 7.4 | 0.00 |
| benzaldehyde | HBA | 1.06 | 2.25 | | 1.48 | | 9.8 | 0.00 |
| 2-chloropyridine | HBA | 0.65 | 2.00 | | 1.22 | | 9.0 | 0.00 |
| methyl benzoate | HBA | 1.56 | 2.80 | | 2.12 | | 12.4 | 0.00 |
| triethylamine | HBA | 0.72 | 1.86 | | 1.45 | | 9.7 | 0.00 |
| 2-methylpyrazine | HBA | -0.79 | 1.04 | | 0.23 | | 8.8 | 0.02 |
| acetpphenone | HBA | 1.14 | 2.79 | | 1.58 | | 11.4 | 0.04 |
| ethyl phenyl ether | HBA | 2.61 | 3.62 | | 2.51 | | 12.0 | 0.05 |
| methyl phenyl ether | HBA | 2.09 | 3.12 | | 2.11 | | 10.4 | 0.07 |
| 1-chloropropane | HBA | 1.96 | 2.46 | | 2.04 | | 6.9 | 0.09 |
| iodomethane | HBA | 1.46 | 2.13 | | 1.51 | | 4.5 | 0.12 |
| chloromethane | HBA | 0.76 | 1.42 | | 0.91 | | 3.7 | 0.12 |
| bromoethane | HBA | 1.58 | 2.24 | | 1.61 | | 5.7 | 0.12 |
| diethyl sulfide | HBA | 2.03 | 3.64 | | 1.95 | | 9.0 | 0.14 |
| 1,1,1-trichloroethane | HBA | 2.59 | 3.10 | | 2.49 | | 7.7 | 0.16 |
| 1,4-dioxane | HBA | -0.81 | 0.73 | | -0.27 | | 8.4 | 0.19 |
| methyl hexanoate | HBA | 2.04 | 3.48 | | 2.42 | | 13.2 | 0.23 |
| Solute | Type* | logP_16_ | logP_chl_ | | logP_oct_ | | S_m_ | H_M_HBD_ |
| methyl pentanoate | HBA | 1.51 | 3.01 | | 1.96 | | 11.6 | 0.23 |
| n-pentyl acetate | HBA | 2.00 | 3.60 | | 2.30 | | 13.2 | 0.23 |
| n-butyl acetate | HBA | 1.41 | 3.05 | | 1.78 | | 11.6 | 0.23 |
| n-propyl acetate | HBA | 0.77 | 2.56 | | 1.24 | | 10.0 | 0.24 |
| methyl propate | HBA | 0.28 | 1.87 | | 0.82 | | 8.4 | 0.24 |
| ethyl acetate | HBA | 0.15 | 1.82 | | 0.73 | | 8.4 | 0.24 |
| Butanone | HBA | -0.43 | 1.15 | | 0.29 | | 7.4 | 0.25 |
| methyl acetate | HBA | -0.39 | 1.16 | | 0.18 | | 6.8 | 0.27 |
| methyl phenyl sulfone | HBA | -0.92 | 1.93 | | 0.50 | | 13.4 | 0.35 |
| propanone | HBA | -1.09 | 0.50 | | -0.24 | | 5.8 | 0.40 |
| trimethylamine | HBA | -0.73 | 0.51 | | 0.22 | | 6.7 | 0.48 |
| acetonitrile | HBA+HBD | -1.11 | 0.40 | | -0.34 | | 3.9 | 0.42 |
| nitromethane | HBA+HBD | -1.06 | 0.44 | | -0.35 | | 4.9 | 0.45 |
| 1,2-dichloroethane | HBA+HBD | 1.26 | 2.13 | | 1.48 | | 6.8 | 0.53 |
| 1,1-dichloroethane | HBA+HBD | 1.70 | 2.39 | | 1.79 | | 6.6 | 0.74 |
| dichloromethane | HBA+HBD | 1.06 | 2.00 | | 1.25 | | 5.2 | 0.78 |
| 1,1,2-trichloroethane | HBA+HBD | 1.83 | 2.41 | | 1.89 | | 8.1 | 1.69 |
| diisopropylamine | HBA+HBD | 0.53 | 1.61 | | 1.16 | | 11.1 | 2.17 |
| diethylamine | HBA+HBD | -0.60 | 0.79 | | 0.58 | | 8.3 | 2.21 |
| dimethylamine | HBA+HBD | -1.55 | -0.44 | | -0.38 | | 5.1 | 2.25 |
| trichloromethane | HBA+HBD | 1.69 | 2.28 | | 1.97 | | 6.5 | 2.53 |
| n-butylamine | HBA+HBD | -0.49 | 0.75 | | 0.97 | | 8.3 | 3.99 |
| n-propylamine | HBA+HBD | -1.08 | 0.25 | | 0.48 | | 6.7 | 4.00 |
| ethylamine | HBA+HBD | -1.62 | -0.35 | | -0.13 | | 5.1 | 4.01 |
| methylamine | HBA+HBD | -2.04 | -1.02 | | -0.57 | | 3.5 | 4.05 |
| 2-methylpropan-2-ol | HBA+HBD | -1.32 | -0.02 | | 0.35 | | 7.4 | 6.16 |
| cyclohexan-ol | HBA+HBD | -0.25 | 1.12 | | 1.23 | | 10.4 | 6.32 |
| butan-2-ol | HBA+HBD | -1.05 | 0.30 | | 0.61 | | 7.8 | 6.38 |
| propan-2-ol | HBA+HBD | -1.72 | -0.35 | | 0.05 | | 6.2 | 6.43 |
| 2-methylpropan-1-ol | HBA+HBD | -0.89 | 0.34 | | 0.76 | | 7.8 | 6.46 |
| pentan-1-ol | HBA+HBD | -0.24 | 1.05 | | 1.56 | | 9.6 | 6.48 |
| hexan-1-ol | HBA+HBD | 0.38 | 1.69 | | 2.03 | | 11.2 | 6.48 |
| heptan-1-ol | HBA+HBD | 1.03 | 2.41 | | 2.72 | | 12.8 | 6.48 |
| butan-1-ol | HBA+HBD | -0.86 | 0.42 | | 0.88 | | 8.0 | 6.49 |
| propan-1-ol | HBA+HBD | -1.53 | -0.30 | | 0.25 | | 6.4 | 6.50 |
| ethanol | HBA+HBD | -2.19 | -0.87 | | -0.30 | | 4.8 | 6.55 |
| methanol | HBA+HBD | -2.77 | -1.33 | | -0.74 | | 3.2 | 6.68 |
| 3-methylbutanoic acid | HBA+HBD | -1.33 | 0.19 | | 1.16 | | 9.8 | 10.73 |
| hexanoic acid | HBA+HBD | -0.64 | 1.02 | | 1.92 | | 11.6 | 10.76 |
| Solute | Type* | logP_16_ | logP_chl_ | | logP_oct_ | | S_m_ | H_M_HBD_ |
| pentanoic acid | HBA+HBD | -1.14 | 0.32 | | 1.39 | | 10.0 | 10.77 |
| butanoic acid | HBA+HBD | -1.83 | -0.27 | | 0.79 | | 8.4 | 10.79 |
| 2-methylphenol | HBA+HBD | -0.09 | 1.23 | | 1.98 | | 10.4 | 10.83 |
| propanoic acid | HBA+HBD | -2.45 | -0.86 | | 0.33 | | 6.8 | 10.86 |
| 4-methylphenol | HBA+HBD | -0.19 | 1.06 | | 1.97 | | 10.4 | 10.98 |
| acetic acid | HBA+HBD | -3.16 | -1.46 | | -0.17 | | 5.2 | 11.04 |
| acetamide | HBA+HBD | -4.68 | -1.97 | | -1.26 | | 5.5 | 11.27 |
| phenol | HBA+HBD | -1.08 | 0.32 | | 1.46 | | 8.8 | 11.80 |
| 3-methoxyphenol | HBA+HBD | -1.08 | 0.77 | | 1.58 | | 11.4 | 12.43 |
| 4-bromophenol | HBA+HBD | -0.10 | 1.07 | | 2.59 | | 11.0 | 13.43 |
| 4-chlorophenol | HBA+HBD | -0.75 | 1.07 | | 2.40 | | 10.5 | 13.43 |
| 3-nitrophenol | HBA+HBD | -1.37 | 0.50 | | 2.00 | | 11.5 | 16.83 |
| 4-nitrophenol | HBA+HBD | -1.93 | 0.20 | | 1.91 | | 11.5 | 17.32 |

^*^Compounds and their experimental logP_16_, logP_chl_ and logP_oct_ values are collected from references.([Abraham et al., 1994](#_ENREF_1); [Abraham et al., 1999](#_ENREF_4)) Only the compounds that have all the experimental logP_16_, logP_chl_ and logP_oct_ data and do not have intramolecular H-bonds are listed in this table.

**Supplementary Table 2:** Experimental and calculated values of logK_p_ (the logarithm of experimental human skin permeability) for neutral organic compounds and the solute descriptors used for this work. K_p_ is in units of cm/s;

|  | Solute | H_M_HBD_ | S_m_ | Flex | logP_oct_ | | | logK_p_ (obs) | logKp (calc)^$^ | |
| --- | --- | --- | --- | --- | --- | --- | --- | --- | --- | --- |
|  |  |  |  |  | (obs) | | (calc)^@^ |  |  |  |
| 1 | 2-ethoxyethanol | 7.15 | 9.0 | 4.0 | -0.10 | | -0.05 | -6.68^#^ | -6.44 |  |
| 2 | formic acid | 11.20 | 3.6 | 0.0 | -0.54 | | -0.73 | -6.60^&^ | -6.59 |  |
| 3 | methanol | 6.68 | 3.2 | 0.0 | -0.74 | | -0.72 | -6.38^#^ | -6.45 |  |
| 4 | hydroquinone | 18.90 | 9.8 | 0.0 | 0.59 | | 0.49 | -6.31^#^ | -6.32 |  |
| 5 | propanoic acid | 10.86 | 6.8 | 1.0 | 0.33 | | 0.20 | -6.02^&^ | -6.10 |  |
| 6 | butanoic acid | 10.79 | 8.4 | 2.0 | 0.79 | | 0.74 | -5.98^&^ | -5.89 |  |
| 7 | cetechol* | 12.57* | 9.8 | 0.0 | 0.88 | | 0.47 | -5.87^#^ | -5.75 |  |
| 8 | Diethyl ether | 0.00 | 8.0 | 3.0 | 0.89 | | 1.25 | -5.37^#^ | -5.27 |  |
| 9 | pentanoic acid | 10.77 | 10.0 | 3.0 | 1.39 | | 1.30 | -5.66^&^ | -5.59 |  |
| 10 | 4-cyanophenol | 16.47 | 10.5 | 0.0 | 1.60 | | 1.35 | -5.53^#^ | -5.52 |  |
| 11 | 3-nitrophenol | 16.83 | 11.5 | 0.0 | 2.00 | | 1.83 | -5.33^#^ | -5.28 |  |
| 12 | 4-nitrophenol | 17.32 | 11.5 | 0.0 | 1.91 | | 1.77 | -5.33^#^ | -5.36 |  |
| 13 | benzyl alcohol | 7.33 | 10.4 | 0.5 | 1.10 | | 1.17 | -5.30^#^ | -5.34 |  |
| 14 | phenol | 11.80 | 8.8 | 0.0 | 1.46 | | 1.59 | -5.27^#^ | -5.32 |  |
| 15 | benzoic acid | 11.80 | 10.8 | 0. 0 | 1.87 | | 1.87 | -5.15^&^ | -5.06 |  |
| 16 | hexan-1-ol | 6.48 | 11.2 | 4.0 | 2.03 | | 1.99 | -4.92^#^ | -5.01 |  |
| 17 | 3-methylphenol | 11.32 | 10.4 | 0.0 | 1.98 | | 2.01 | -4.89^#^ | -4.96 |  |
| 18 | 4-methylphenol | 10.98 | 10.4 | 0.0 | 1.97 | | 2.01 | -4.83^&^ | -4.94 |  |
| 19 | heptanoic acid | 10.76 | 13.2 | 5.0 | 2.41 | | 2.40 | -4.79^&^ | -5.12 |  |
| 20 | methyl phenyl ether | 0.07 | 10.4 | 1.5 | 2.11 | | 2.28 | -4.68^#^ | -4.34 |  |
| 21 | 2-naphthol | 11.56 | 13.4 | 0.0 | 2.70 | | 2.79 | -4.65^#^ | -4.50 |  |
| 22 | 2-chlorophenol* | 6.83* | 10.3 | 0.0 | 2.15 | | 2.36 | -4.56^#^ | -4.58 |  |
| 23 | 4-ethylphenol | 10.66 | 12.0 | 0.5 | 2.58 | | 2.42 | -4.53^#^ | -4.58 |  |
| 24 | 3,4-dimethylphenol | 10.53 | 12.0 | 0.5 | 2.23 | | 2.35 | -4.52^&^ | -4.75 |  |
| 25 | benzaldehyde | 0.00 | 9.8 | 0.0 | 1.48 | | 1.48 | -4.51^#^ | -4.60 |  |
| 26 | 4-butylphenol | 10.59 | 15.2 | 2.5 | 3.56 | | 3.52 | -4.47^#^ | -4.12 |  |
| 27 | octan-1-ol | 6.48 | 14.4 | 6.0 | 3.07 | | 3.09 | -4.30^#^ | -4.53 |  |
| 28 | tribromomethane | 3.19 | 7.7 | 0.0 | 2.67 | | 2.40 | -4.34^#^ | -4.02 |  |
| 29 | nonan-1-ol | 6.48 | 16.0 | 7.0 | 3.67 | | 3.64 | -4.30^#^ | -4.23 |  |
| 30 | 2-nitrophenol* | 1.25* | 11.5 | 0.0 | 1.85 | | 1.70 | -4.08^&^ | -4.44 |  |
| 31 | methylbenzene | 0.00 | 9.4 | 0.0 | 2.73 | | 2.64 | -3.64^#^ | -3.79 |  |
| 32 | 2-phenylethanol | 6.80 | 12.0 | 1.5 | 1.36 | | 1.30 | -5.20^#^ | -5.23 |  |
| 33 | decan-1-ol | 6.48 | 17.6 | 8.0 | 3.91^%^ | | 3.91 | -4.15^#^ | -4.15 |  |
| 34 | 4-chloro-3-methylphenol | 12.93 | 11.9 | 0.0 | 2.84^%^ | | 2.84 | -4.34^#^ | -4.50 |  |
| 35 | resorcinol | 23.46 | 9.8 | 0.0 | 0.80 | | 0.97 | -6.70^#^ | -6.46 |  |
| 36 | acetic acid | 11.04 | 5.2 | 0.0 | -0.17 | | -0.33 | -6.53^&^ | -6.34 |  |
|  |  |  |  |  |  |  | |  |  |  |
|  | Solute | H_M_HBD_ | S_m_ | Flex | logP_oct_ | | | logK_p_ (obs) | logKp (calc)^$^ |  |
|  |  |  |  |  | (obs) | | (calc) ^@^ |  |  |  |
| 37 | ethanol | 6.55 | 4.8 | 0.0 | -0.30 | -0.19 | | -6.08^#^ | -6.15 |  |
| 38 | propan-1-ol | 6.50 | 6.4 | 1.0 | 0.25 | 0.34 | | -5.93^#^ | -5.89 |  |
| 39 | butan-1-ol | 6.49 | 8.0 | 2.0 | 0.88 | 0.89 | | -5.70^#^ | -5.57 |  |
| 40 | Butanone | 0.25 | 7.4 | 1.0 | 0.29 | 0.44 | | -5.42^#^ | -5.48 |  |
| 41 | pentan-1-ol | 6.48 | 9.6 | 3.0 | 1.56 | 1.44 | | -5.30^#^ | -5.22 |  |
| 42 | hexanoic acid | 10.76 | 11.6 | 4.0 | 1.92 | 1.85 | | -4.94^&^ | -5.34 |  |
| 43 | 2-methylphenol | 10.83 | 10.4 | 0.0 | 1.98 | 2.00 | | -4.88^&^ | -4.93 |  |
| 44 | octanoic acid | 10.76 | 14.8 | 6.0 | 3.05 | 2.96 | | -4.70^&^ | -4.80 |  |
| 45 | heptan-1-ol | 6.48 | 12.8 | 5.0 | 2.72 | 2.54 | | -4.57^#^ | -4.66 |  |
| 46 | trichloromethane | 2.53 | 6.5 | 0.0 | 1.97 | 2.01 | | -4.46^#^ | -4.44 |  |
| 47 | Methyl paraben | 14.99 | 12.4 | 0.5 | 1.96 | 1.76 | | -5.03^#^ | -5.24 |  |
| 48 | 4-chlorophenol | 13.43 | 10.3 | 0.0 | 2.40 | 2.39 | | -4.52^#^ | -4.81 |  |
| 49 | 4-bromophenol | 13.43 | 10.7 | 0. 0 | 2.59 | 2.48 | | -4.52^#^ | -4.69 |  |
| 50 | benzene | 0.00 | 7.8 | 0.0 | 2.13 | 2.13 | | -4.27^#^ | -4.18 |  |
| 51 | ethylbenzene | 0.00 | 11.0 | 0.5 | 3.15 | 3.11 | | -3.00^#^ | -3.56 |  |

^#^ Experimental logK_p_ values are taken from a paper of Abraham, M. H.’s group.([Zhang et al., 2017](#_ENREF_7))

^&^ Experimental logK_p_ values are taken from another paper of Abraham, M. H.’s group.([Abraham and Martins, 2004](#_ENREF_3))

^$^ The values are calculated from the model: logK_p_ =0.6501 logP_oct_(exp) – 0.0602 H_M_HBD_ -0.0947*Flex + -5.563. This model is derived from the data for compounds 1-32.

^*^Because the polar hydrogen atoms of the molecules form intramolecular H-bonds, the H_M_HBD_ values are calculated from the formula H_M_HBD_ = 0.822 H_M_ – 1.188$H_{M}^{\mathrm{oct}}$ + 1.251 (see a reference([Chen et al., 2020](#_ENREF_6)) for more details).

^%^ The logP_oct_ values are calculated from the logP_16_ values based on the model logP_oct_ = 0.7910 logP_16_ + 0.1585 H_M_HBD_ + 0.545.

^@^ The logP_oct_ values are calculated from the structures of the solutes, based on the model developed in a previous study.([Chen et al., 2020](#_ENREF_6))

**Supplementary Table 3:** Experimental and calculated values of logK_brain_ for volatile organic compounds and the logP_16_, logP_oct_ and S_m_ values of the compounds.^&^

| Solute | logP_oct_ | logP_16_ | S_m_ | H_M_HBD_ | Flex | log_brainl_ | logK_brain_^#^  (calc)_LOO_ | logK_brain_^$^ (calc_)Eq_ |
| --- | --- | --- | --- | --- | --- | --- | --- | --- |
|  |  |  |  |  |  | (obs) |  |  |
| Neon | 0.28 | 0.38 | 0.4 | 0.00 | 0.0 | -1.81 | -1.60 | -1.62 |
| argon | 0.74 | 0.78 | 1.1 | 0.00 | 0.0 | -1.49 | -1.49 | -1.53 |
| krypton | 0.89 | 1.00 | 1.8 | 0.00 | 0.0 | -1.38 | -1.24 | -1.26 |
| xenon | 1.28 | 1.35 | 2.5 | 0.00 | 0.0 | -0.70 | -1.12 | -1.15 |
| nitrogen |  | 0.82 | 1.4 | 0.00 | 0.0 | -1.80 | -1.30 | -1.32 |
| methane | 1.09 | 1.14 | 2.2 | 0.00 | 0.0 | -1.39 | -1.10 | -1.12 |
| n-pentane | 3.39 | 3.87 | 8.6 | 0.00 | 2.0 | 0.34 | 0.30 | 0.26 |
| n-hexane | 3.90 | 4.49 | 10.2 | 0.00 | 3.0 | 0.70 | 0.58 | 0.54 |
| 2-methylpentane |  | 4.34 | 10.0 | 0.00 | 2.0 | 0.58 | 0.77 | 0.75 |
| 3-methylpentane | 3.60 | 4.42 | 10.0 | 0.00 | 2.0 | 0.64 | 0.72 | 0.69 |
| 2,2-dimethylbutane | 3.82 | 4.19 | 9.6 | 0.00 | 1.0 | 0.45 | 0.86 | 0.84 |
| n-heptane | 4.50 | 5.14 | 11.8 | 0.00 | 4.0 | 1.09 | 0.82 | 0.77 |
| 3-methylHexane |  | 5.03 | 11.6 | 0.00 | 3.0 | 1.01 | 1.02 | 0.98 |
| n-octane | 5.15 | 5.79 | 13.4 | 0.00 | 5.0 | 1.37 | 1.05 | 1.38 |
| methylcyclopentane | 3.37 | 3.99 | 9.4 | 0.00 | 0.0 | 0.86 | 1.05 | 0.99 |
| cyclohexane | 3.44 | 3.91 | 9.6 | 0.00 | 0.0 | 1.04 | 1.18 | 1.20 |
| cyclopropane | 1.72 | 1.86 | 4.8 | 0.00 | 0.0 | -0.10 | -0.20 | -0.18 |
| ethene | 1.13 | 1.23 | 3.2 | 0.00 | 0.0 | -0.26 | -0.70 | -0.69 |
| propene | 1.77 | 1.92 | 4.8 | 0.00 | 0.0 | -0.22 | -0.22 | -0.21 |
| but-1,3-diene | 1.99 | 1.99 | 5.8 | 0.00 | 1.0 | -0.08 | 0.06 | 0.10 |
| dichloromethane | 1.25 | 1.06 | 5.6 | 0.78 | 0.0 | 0.78 | 0.48 | 0.53 |
| trichloromethane | 1.97 | 1.69 | 6.5 | 2.53 | 0.0 | 1.30 | 0.97 | 0.83 |
| 1,1,1-trichloroethane | 2.49 | 2.59 | 7.7 | 0.16 | 0.0 | 0.92 | 0.90 | 0.96 |
| trichloroethene | 2.42 | 2.68 | 7.7 | 2.17 | 0.0 | 1.33 | 1.02 | 0.86 |
| diethyl ether | 0.89 | 0.85 | 8.0 | 0.00 | 3.0 | 1.10 | 1.47 | 1.74 |
| propanone | -0.24 | -1.09 | 5.8 | 0.40 | 0.0 | 2.19 | 1.71 | 2.20 |
| Butanone | 0.29 | -0.43 | 7.4 | 0.25 | 1.0 | 2.07 | 2.14 | 2.52 |
| ethanol | -0.30 | -2.19 | 4.8 | 6.55 | 0.0 | 3.02 | 2.39 | 2.19 |
| propan-1-ol | 0.25 | -1.53 | 6.4 | 6.50 | 1.0 | 2.87 | 2.79 | 2.55 |
| propan-2-ol | 0.05 | -1.72 | 6.4 | 6.43 | 0.0 | 2.76 | 3.06 | 2.81 |
| 2-methylpropan-1-ol | 0.76 | -0.89 | 7.8 | 6.46 | 1.0 | 2.61 | 3.36 | 3.04 |
| benzene | 2.13 | 2.15 | 7.8 | 0.00 | 0.0 | 1.26 | 1.15 | 1.28 |
| methylbenzene | 2.73 | 2.68 | 9.4 | 0.00 | 0.0 | 1.56 | 1.71 | 1.86 |
| Carbon dioxide |  | 0.14 | 3.0 | 0.00 | 0.0 | -0.25 | -0.21 | -0.08 |

^&^All logP_oct_ and logP_16_ data are experimental data. Experimental logK_brain_ values are taken from a reference([Abraham et al., 2006](#_ENREF_2)).

^#^logK_brain_ values are calculated from the LFER model with logP_16_, S_m_, H_M_HBD_ and Flex as predictive variables by using leave-one-out (LOO) cross-validation technique.

^$^ logK_brain_ values are calculated from the LFER model with logP_16_, S_m_ and Flex as predictive variables by using leave-one-out (LOO) cross-validation technique.

**Supplementary Table 4.** Experimental and calculated values of logP_aln_ (the logarithm of the partition coefficient between aniline and water) and the solute descriptors used for this work;

| Solute | P_16_ | S_m_ | logP_aln_ (obs)^&^ | logP_aln_ (calc)^$^ |
| --- | --- | --- | --- | --- |
| hydrogen | 0.52 | 0.6 | 0.175 | 0.345 |
| xenon | 1.35 | 2.5 | 0.693 | 1.021 |
| radon | 1.52 | 3.1 | 1.194 | 1.191 |
| nitrogen | 0.82 | 1.4 | 0.356 | 0.606 |
| oxygen | 0.79 | 1.4 | 0.290 | 0.592 |
| 3-methylbut-1-ene | 3.25 | 7.8 | 2.798 | 2.711 |
| 2-methylpent-1-ene | 3.62 | 9.6 | 3.045 | 3.156 |
| 2,2,4-trimethylpentane | 5.28 | 12.6 | 4.179 | 4.387 |
| 2-methylbutane | 3.76 | 8.4 | 2.880 | 3.041 |
| methane | 1.14 | 2.2 | 0.688 | 0.877 |
| ethane | 1.83 | 3.8 | 1.505 | 1.442 |
| propane | 2.49 | 5.4 | 2.019 | 1.993 |
| butane | 3.13 | 7.0 | 2.544 | 2.534 |
| 2-methylpropane | 3.11 | 6.8 | 2.488 | 2.495 |
| pentane | 3.87 | 8.6 | 3.024 | 3.123 |
| hexane | 4.49 | 10.2 | 3.526 | 3.655 |
| heptane | 5.14 | 11.8 | 4.143 | 4.201 |
| octane | 5.79 | 13.4 | 4.695 | 4.747 |
| cyclopentane | 3.40 | 8.0 | 2.751 | 2.812 |
| cyclohexane | 3.91 | 9.6 | 3.109 | 3.292 |
| methylcyclohexane | 4.49 | 11.0 | 3.679 | 3.775 |
| ethene | 1.23 | 3.2 | 1.049 | 1.070 |
| propene | 1.92 | 4.8 | 1.759 | 1.635 |
| 1-pentene | 3.28 | 8.0 | 2.741 | 2.755 |
| 1-hexene | 3.73 | 9.6 | 3.115 | 3.207 |
| 1-heptene | 4.28 | 11.2 | 3.606 | 3.707 |
| 1-octene | 4.98 | 12.8 | 4.203 | 4.276 |
| cyclohexene | 3.29 | 9.6 | 2.830 | 3.001 |
| 1,3-butadiene | 1.99 | 5.8 | 1.962 | 1.818 |
| dichloromethane | 1.06 | 5.2 | 1.562 | 1.291 |
| trichloromethane | 1.69 | 6.5 | 2.052 | 1.783 |
| tetrachloromethane | 2.88 | 7.6 | 2.641 | 2.507 |
| chloroethane | 1.22 | 5.3 | 1.481 | 1.381 |
| 1-chloropropane | 1.96 | 6.9 | 2.060 | 1.970 |
| 1-chlorobutane | 2.60 | 8.5 | 2.684 | 2.511 |
| 2-chloro-2-methylpropane | 3.02 | 7.9 | 2.946 | 2.618 |
|  |  |  |  |  |
| Solute | P_16_ | S_m_ | logP_aln_ (obs)^&^ | logP_aln_ (calc) ^$^ |
| ethyl bromide | 1.58 | 5.7 | 1.763 | 1.611 |
| methyl iodide | 1.46 | 4.5 | 1.599 | 1.373 |
| ethyl iodide | 2.03 | 6.1 | 2.187 | 1.882 |
| diethyl ether | 0.85 | 8.0 | 1.487 | 1.614 |
| 1,4-dioxane | -0.81 | 8.4 | 0.459 | 0.895 |
| ethyl acetate | 0.15 | 8.4 | 1.023 | 1.346 |
| propanone | -1.09 | 5.8 | 0.201 | 0.372 |
| butanone | -0.43 | 7.4 | 0.847 | 0.923 |
| nitromethane | -1.06 | 4.9 | 0.737 | 0.250 |
| methanol | -2.77 | 3.2 | -0.912 | -0.808 |
| ethanol | -2.19 | 4.8 | -0.517 | -0.295 |
| 1-butanol | -0.86 | 6.4 | 0.487 | 0.570 |
| benzene | 2.15 | 7.8 | 2.358 | 2.194 |
| toluene | 2.68 | 9.4 | 2.750 | 2.684 |
| o-xylene | 3.28 | 11.0 | 3.296 | 3.207 |
| naphthalene | 3.41 | 12.4 | 3.860 | 3.479 |
| chlorobenzene | 2.84 | 9.3 | 2.962 | 2.744 |
| acetonitrile | -1.11 | 3.9 | 0.582 | 0.076 |

^&^ Experimental logK_aln_ values are taken from a reference([Abraham et al., 2015](#_ENREF_5))

^$^ The values are calculated from the model: logP_aln_ = 0.4695 logP_16_ + 0.1506S_m_ + 0.010, which is derived from the data of the 54 compounds in this table.

**References**

Abraham, M.H., Chadha, H.S., Whiting, G.S., and Mitchell, R.C. (1994). Hydrogen bonding. 32. An analysis of water-octanol and water-alkane partitioning and the delta log P parameter of seiler. J Pharm Sci *83*, 1085-1100.

Abraham, M.H., Ibrahim, A., and Acree, W.E., Jr. (2006). Air to brain, blood to brain and plasma to brain distribution of volatile organic compounds: linear free energy analyses. Eur J Med Chem *41*, 494-502.

Abraham, M.H., and Martins, F. (2004). Human Skin Permeation and Partition: General Linear Free-Energy Relationship Analyses. J Pharm Sci *93*, 1508-1522.

Abraham, M.H., Platts, J.A., Hersey, A., Leo, A.J., and Taft, R.W. (1999). Correlation and estimation of gas-chloroform and water-chloroform partition coefficients by a linear free energy relationship method. J Pharm Sci *88*, 670-679.

Abraham, M.H., Zad, M., and Acree, W.E. (2015). The transfer of neutral molecules from water and from the gas phase to solvents acetophenone and aniline. J Mol Liq *212*, 301-306.

Chen, D.L., Wang, Q.Y., Li, Y.B., Li, Y.D., Zhou, H., and Fan, Y.L. (2020). A general linear free energy relationship for predicting partition coefficients of neutral organic compounds. Chemosphere *247*, 125869.

Zhang, K.D., Abraham, M.H., and Liu, X.L. (2017). An equation for the prediction of human skin permeability of neutral molecules, ions and ionic species. Int J Pharmaceut *521*, 259-266.
